# Lung IL-13 gene signatures are associated with raised tissue eosinophils in COPD

**DOI:** 10.1186/s12931-025-03177-x

**Published:** 2025-03-25

**Authors:** Karl J. Staples, Jodie Ackland, Sruthymol Lukose, Bastian Angermann, Graham Belfield, Maria Belvisi, Raghothama Chaerkady, Damla Etal, Ashley Heinson, Sonja Hess, Ventzislava A. Hristova, Michael Hühn, Christopher McCrae, Daniel Muthas, Lisa Öberg, Kristoffer Ostridge, Adam Platt, C. Mirella Spalluto, Alastair Watson, Tom Wilkinson, the MICAII study group

**Affiliations:** 1 Faculty of Medicine, University of Southampton, Southampton, UK; 2 NIHR Southampton Biomedical Research Centre, University Hospital Southampton, Southampton, UK; 3 Translational Science and Experimental Medicine, Research and Early Development, Respiratory & Immunology, BioPharmaceuticals R&D, AstraZeneca, Gothenburg, Sweden; 4 Translational Genomics, Discovery Biology, Discovery Sciences, BioPharmaceuticals R&D, AstraZeneca, Gothenburg, Sweden; 5 Research and Early Development, Respiratory & Immunology, BioPharmaceuticals R&D, AstraZeneca, Gothenburg, Sweden; 6 NHLI, Imperial College, London, UK; 7 Dynamic Omics, Centre of Genomics Research (CGR), Discovery Sciences, BioPharmaceuticals R&D, AstraZeneca, Gaithersburg, USA; 8 Translational Science and Experimental Medicine, Research and Early Development, Respiratory & Immunology, BioPharmaceuticals R&D, AstraZeneca, Gaithersburg, USA; 9 Translational Science and Experimental Medicine, Research and Early Development, Respiratory & Immunology, BioPharmaceuticals R&D, AstraZeneca, Cambridge, UK

**Keywords:** COPD, Exacerbations, Eosinophils

## Abstract

**Background:**

The role of eosinophils in COPD and their utility as biomarkers for cytokine targeting monoclonal therapies remains unclear. We investigated the distribution of eosinophils across different tissue compartments in COPD and analysed gene expression to understand the possible mechanistic drivers of eosinophilic inflammation in COPD.

**Methods:**

Blood and BAL from ex-smoking volunteers with mild/moderate COPD (n = 31) and healthy ex-smoking controls (n = 20), and bronchial biopsy tissue in a subcohort (n = 19 and n = 8, respectively) was analysed. Differentially-expressed genes (DEGs) were characterised using RNASeq. Proteomic analysis of BAL was conducted using mass-spectrometry.

**Results:**

COPD subjects had more eosinophils in blood and lung tissue compared to controls, with increased eosinophil protein CLC/Galectin-10 in BAL. However, peripheral blood eosinophil counts related poorly to numbers in lung tissue (rho = -0.09192, p = 0.3541) or proportions in BAL (rho = 0.01762, p = 0.4632). Tissue IL-5Rα expression was higher in frequent exacerbators and related to tissue eosinophils, but not peripheral blood eosinophils.

Higher blood eosinophils were associated with DEGs that differed with compartment. Higher tissue eosinophil levels were associated with IL-13-induced DEGs including *POSTN* in bronchial brushes and *CCL26* in bronchial biopsies. Gene-set enrichment analysis on data from brushings revealed significant enrichment of IL-4/IL-13, but not IL-5, pathways associated with eosinophil presence.

**Conclusion:**

Eosinophilic lung inflammation is related to exacerbation frequency, but lung eosinophils are not predicted by blood eosinophil counts in COPD. Our data suggest IL-13-mediated pathways may be responsible for the presence of tissue eosinophils in COPD. Further work to establish more predictive biomarkers of lung eosinophil biology are required to unlock this axis to optimised treatment.

**Supplementary Information:**

The online version contains supplementary material available at 10.1186/s12931-025-03177-x.

## Background

Chronic Obstructive Pulmonary Disease (COPD) is now the third leading cause of global mortality [1]. Current treatments remain inadequate, with only modest impacts on morbidity and mortality. The prospect of stratifying patients for certain therapies is attractive and previous reports highlight blood eosinophil counts as a marker of steroid efficacy in COPD (reviewed in [2, 3]). Notably, GOLD recommends using blood eosinophil counts ≥ 300 cells/µl as a biomarker to be used alongside clinical assessment to choose those most likely to benefit from inhaled corticosteroids [4]. Those patients who have blood eosinophil counts ≤ 100 cells/ µl are less likely to benefit from inhaled corticosteroid therapy [4]. The potential of more targeted therapeutics, such as antibodies targeting IL-5 or its receptor, requires further understanding after clinical trials using eosinophil-specific therapies showed disappointing results [2]. However, a positive large scale clinical trial of dupilumab (a monoclonal antibody targeting the IL-4 and IL-13 shared receptor component) has demonstrated reductions in exacerbation frequency [5]. Whilst peripheral blood eosinophil counts appear useful to select patients for dupliumab treatment overall, it remains uncertain how useful this approach was to determine individual clinical response. Indeed the efficacy of this intervention was greater in subjects further stratified by a raised FeNO level, suggesting direct measures of pulmonary inflammation may offer additional benefits.

Previous reports suggest that COPD patients show increased numbers of lung eosinophils compared with healthy controls, even when allergy and asthma are excluded [6–9]. Furthermore, recent work has demonstrated an association between raised blood eosinophils and the development of obstructive lung disease [10]. Eosinophilic inflammation may, therefore, play an important role in COPD immune dysregulation. However, the presence and proportion of lung eosinophils varies considerably across COPD patients [7, 11, 12]. This may reflect differences in disease activity, as there is also an association of eosinophils with COPD exacerbations [13, 14].

Sputum has been used to characterise lung eosinophilia in patients with COPD. However, the wider clinical utility of this approach is limited as sputum analysis is largely restricted to research centres [15]. Therefore, blood eosinophils are used as a surrogate marker to enable patient phenotyping more broadly. Whilst there appear to be strong correlations between blood and sputum eosinophils [13, 14], the association of blood eosinophils with eosinophils in lung tissue and bronchoalveolar lavage (BAL) is unclear. Furthermore, the impact of eosinophils in these different lung compartments on disease measures is not well defined [12, 16]. Understanding the nature of eosinophilic inflammation in the COPD lung itself is a key first step to delivering a step-change in treatment outcomes which is already being achieved in asthma [17].

To address these questions, we investigated the distribution of eosinophils in blood, BAL, and bronchial biopsies from deeply phenotyped COPD patients. Furthermore, we characterised gene expression differences between blood, bronchial biopsies and epithelial brushings and their associations with the presence of eosinophils in these different compartments, aiming to understand the role and possible drivers of increased eosinophils in COPD.

## Methods

### Subjects

**Fig. 1:**
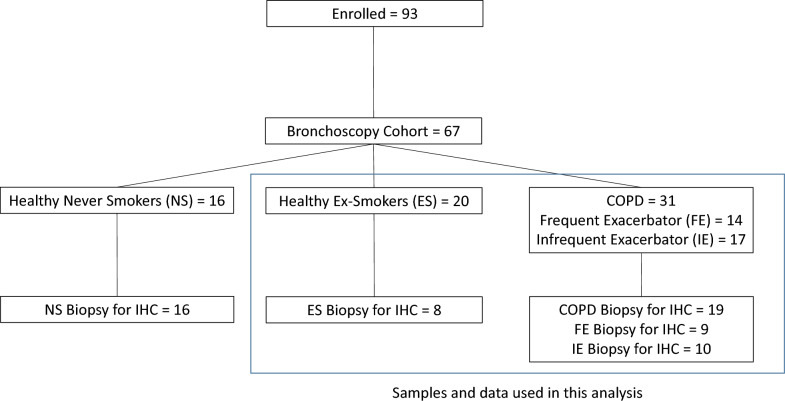
Flow diagram of patient data and samples from the MICAII study. Blue box indicates data and samples used in this analysis

We recruited ex-smoking subjects with mild or moderate COPD (as per GOLD) (n = 31) and healthy ex-smoking volunteers (HV-ES) (n = 20) as the most relevant control group, all with ≥ 10-pack year history and had stopped smoking ≥ 6 months prior to enrolment, as part of the MICAII study (Fig. 1). Patients with a history of asthma or atopy were excluded from the cohort. Additional details about this cohort have been reported previously [18–22]. For additional analyses, COPD subjects were split dependent on exacerbation frequency. COPD subjects were classified as either infrequent exacerbators (IE) (≤ 1 exacerbation in the preceding 12 months before enrolment) or frequent exacerbators (FE) (≥ 2 exacerbations in the preceding 12 months before enrolment). All subjects gave written informed consent, and the study was approved by National Research Ethics Service South Central ethical standards – Hampshire A and Oxford C Committees (LREC no: 15/SC/0528). Sampling was undertaken using fibreoptic bronchoscopy and epithelial brushings, bronchial biopsies and BAL were recovered and processed as previously described [19, 21, 22].

### RNA isolation and sequencing

Total RNA was extracted from epithelial brushing and bronchial biopsy samples using the AllPrep DNA/RNA/miRNA Universal Kit (Qiagen), whilst RNA was extracted from whole blood using the PAXgene Blood RNA Kit (Qiagen). The quantity and quality of RNA samples were determined using the standard RNA analyzer kit on a 96-channel Fragment analyzer (Agilent Technologies). Extracted samples with a yield concentration > 25 ng/µl total RNA, and a DV_200_ value (percentage of RNA fragments > 200nucleotides) >  = 30% were deemed to be of sufficient quantity and quality for TotalRNA-seq analysis. Samples were diluted to 25 ng/µl using a Tecan Fluent liquid handling automation system (Tecan). Library preparation was done in four separate runs, one 96 well plate per run. The Kapa RNA HyperPrep Kit with RiboErase (HMR) was used for reverse transcription, generation of double stranded cDNA and subsequent library preparation and indexing to facilitate multiplexing (Roche), all of which was performed through automation on a Tecan fluent. The libraries were quantified with the 96-channel Fragment Analyzer using the standard sensitivity next generation sequencing (NGS) kit (Agilent Technologies). Samples from each preparation plate were pooled and the final pools (4 in total) were quantified using a Qubit instrument for concentration determination with the DNA High Sensitivity kit (ThermoFisher Scientific). Fragment size was determined using the Fragment Analyzer, standard sensitivity NGS kit (Agilent Technologies). Three of four library pools were further diluted to 1 nM and sequenced on a NovaSeq 6000 (Illumina) using NovaSeq 6000 S4 Reagent Kit, 2 × 76 cycles. The remaining library pool was diluted to 1.9 nM and sequenced on NovaSeq 6000 (Illumina) using 2 NovaSeq 6000 SP S1 Reagent Kits, 2 × 51 cyclers. Average reads per sample were 53.3 million.

### RNASeq analysis

Fastq files from 307 paired-end sequencing libraries generated from 120 epithelial brushings, 125 bronchial biopsies, and 62 blood samples were collected and read quality for all libraries was accessed using FastQC (v0.11.9) [23], Qualimap (v2.2.2d) [24] and samtools stats (v1.15) [25]. Quality control (QC) metrics for Qualimap were based on a STAR (v2.7.10a) [26] alignment against the human genome (GRCh38, Gencode v43). Next, QC metrics were summarized using MultiQC (v1.12) [27]. Two libraries were excluded; one due to a low mapping rate (57% vs [79%−97%]) and another due to low sequencing throughput (210 k reads vs [20 M-86 M]), leaving 118 epithelial brushings, 125 bronchial biopsies, and 62 blood samples for further analysis. Sequencing adapters were then trimmed from the remaining libraries using NGmerge (v0.3) [28]. A human transcriptome index consisting of cDNA and ncRNA entries from Gencode (v43) was generated and reads were mapped to the index using Salmon (v1.7.0) [29]. The bioinformatics workflow was organized using Nextflow workflow management system (v20.10) [30] and Bioconda software management tool [31].

Differential gene expression were assessed with DESeq2 (v 1.34.0) [32], using ashr (v2.2_54) [33] for fold change shrinkage, all in R (v4.1.3) [34]. Estimated counts from Salmon were used as input for DESeq2 (v1.34.0) using tximport (v1.22.0) [35] in R (v4.1.3). In the models used to assess differential expression between subject groups, effects from gender and a technical batch-effect (library prep plate) were taken into account. To ensure that the identified DEGs had robust and biologically meaningful expression, we applied a threshold requiring a median expression value of > 0.5 log2TPM in at least one comparison group, alongside an adjusted p-value < 0.05 determined by the Benjamini–Hochberg multiple testing correction method. DEGs were visualised using the EnhancedVolcano package (v 1.20.0) and DEG overlaps visualised using ggvenn (0.1.10).

The clusterProfiler [36] package (v4.10.0) was used to perform Over Representation Analysis (ORA) and Gene Set Enrichment Analysis (GSEA) on the DEG lists. ORA was performed using enrichGO() for gene ontology (GO) categories Biological Process (BP), Molecular Function (MF) and Cellular Component (CC), enrichKEGG() for KEGG pathways and enrichPathway() for REACTOME pathways. GO categories and KEGG pathways were obtained from Bioconductor org package org.Hs.eg.db (v 3.18.0) [37], and REACTOME pathways were obtained from ReactomePA (version 1.46.0) [37, 38]. All genes tested for DEG analysis were used as the background gene set, min gene set size was 5 and max gene set size was 500. The Benjamini–Hochberg multiple testing correction method was applied, and significant terms/pathways were filtered according to an adjusted p-value < 0.05. The top 5 significant terms/pathways for the ORA were visualised in R using ggplot2 (v 3.4.4).

GSEA was performed using GSEA() on the brushings dataset to further investigate the presence of IL-13 signalling in this sample type. All genes were ranked taking into account both the log2FC (ashr shrunken) and p-value. The INTERLEUKIN_4_AND_INTERLEUKIN_13_SIGNALING curated C2 pathway (R-HSA-6785807) was downloaded using msigdbr (v 7.5.1). Result visualisation was performed using gseaplot2() from the enrichplot package (v 1.22.0).

### Proteomics

Proteins in BAL supernatants were analysed using LC MS/MS as previously described [22]. LC–MS/MS analysis of TMT labelled peptides was carried out on a Q Exactive HF-X (Thermo Fisher Scientific) mass spectrometer interfaced with a Dionex 3000 RSLCnano system. Peptides were captured on a 2 cm × 75 µm C18 trap column (ReproSil-Pur 120 C18-AQ 7um) and samples were separated on a monolithic column (50 cm, cut from a 2 m long column, 100 µm ID, GL Sciences Inc. USA) using a gradient of solvent A (0.2% formic acid) and solvent B (0.2% formic acid in 90% acetonitrile). Peptides were separated using a 90 min gradient of solvent B as follows: 4% to 16.5%B in 2.5—52.5 min; 33.5% B in 73 min followed by a stay at 98% B for 3 min and re-equilibration at 2% B. A flowrate of 0.7 µL/min was used. Peptides were sprayed in an electrospray ionization (ESI) source using a stainless-steel emitter with 2 kV at a capillary temperature of 275 °C. A full-scan MS spectrum was collected at 60,000 resolution at m/z of 200 and scanned at 350–1200 m/z with automatic gain control (AGC) of 3E6. The top 10 precursors were selected, and an MS/MS scan was obtained at 45,000 resolution with 50 ms injection time, isolation window of 0.9 m/z with offset 0.1 m/z, normalized collision energy (NCE) of 29. For MS2, minimum AGC target was set to 1.7E4. Dynamic exclusion duration was set to 15 s. The fixed first mass was set to 100 m/z. Charge state exclusion was set to ignore unassigned, 1, and 7 and greater charges. For internal mass calibration, lock mass of 371.10124 m/z was used.

Mass spectrometry data was analysed using Proteome Discoverer 2.3 (Thermo Fisher Scientific) software with search engine Mascot (version 2.6.0). Data was searched using latest UniProt Human protein database. Unfragmented precursor and TMT reporter ions were removed using a non-fragment filter in the PD 2.3 workflow. Search parameters included 3 missed cleavages for trypsin, oxidation (M) and deamidation (N, Q) as variable modifications. Tandem label (229.163 Da) at N-terminus and lysine residues and carbamidomethylation on cysteine residues were set as fixed modifications. The mass tolerances on precursor and fragment masses were set at 20 ppm and 0.05 Da, respectively, for MS2 analysis. Consensus step in PD2.3 included several nodes for spectrum, peptide and protein grouping and FDR calculation. Reporter ions for TMT labelled peptides were quantified using the PD quantitation node and peak integration tolerance was set at 20 ppm by considering most confident centroid peaks. Signal to noise values were calculated in addition to measurement of intensities of the TMT reporter ion for peptide and protein quantitation. The intensities were normalized by total peptide amount in PD 2.3. To account for protein input, the global quantitative proteome data was reviewed before normalization and no samples showed an unexpected pattern of distribution. Albumin and haemoglobin abundances were not significantly different between sub-cohorts. Further normalization of the data across all samples was carried out using Reporter Ion Quantitation in Proteome Discoverer, which calculates the total sum of the abundance values for each TMT channel over all peptides identified within a file. The channel with the highest total abundances served as a reference for correcting abundances across the remaining channels by a constant factor.

### Immunofluorescence

Biopsies were only available from 19 COPD and 8 HV-ES for imaging analysis as samples for RNASeq analysis was prioritised (Fig. 1). Biopsies for immunofluorescence were fixed in 4% (w/v) formaldehyde and embedded in paraffin wax, as previously prescribed [39, 40]. Ten serial sections of 5 μm thickness were obtained using a Leica RM2135 microtome (Leica Biosystems, Germany). The sections were then mounted onto APES (3-aminopropyltriethoxysilane) coated microscope slides. (CellPath, Powys). To deparaffinise the tissue, microscope slides containing the tissue were placed into slide racks and into clearene (Leica Biosystems) twice with ten minutes each in a clearene tank. The slides were rehydrated by soaking them in graded alcohols solutions of 100% ethanol, 75% ethanol and 70% ethanol, 5 min each.

To perform H&E stains, histology slides were placed into Mayer’s Haematoxylin (CellPath, Powys) solution for 5 min, then placed under running tap water for 5 min. The slides were then placed into eosin stain (CellPath, Powys) for further 5 min. Slides were then briefly placed into 100% ethanol, after which they were dehydrated by taking them into 95% ethanol and 100% ethanol (1 min on each). After which they were then placed into clearene three times (3 min each). The slides were mounted in XFT mounting medium (CellPath, Powys) and cover slipped.

Antigen retrieval was performed by pipetting 5% (v/v) pronase solution onto the tissue. These slides were left to incubate at room temperature for 10 min, and then rinsed in 1 × PBS. The tissue slides were incubated at room temperature for a further 60 min with blocking solution (1 × PBS + 1% bovine serum albumin & 2% foetal calf serum). Excess blocking buffer was removed, primary antibodies were added and incubated overnight at 4 °C. Primary antibodies were added to all slides except the negative controls. 0.00125 mg/ml mouse anti-EG2 (Diagnostic Development, Uppsala, Sweden) or 0.005 mg/ml rabbit anti-IL-5Rα (ThermoFisher, UK) primary antibodies incubated overnight at 4 °C. Tissue was also incubated overnight with just the blocking buffer. After overnight incubation, the slides were washed in PBS and then 0.002 mg/ml AlexaFluor647 goat anti-mouse or 0.004 mg/ml AlexaFluor647 goat anti-rabbit secondary antibodies (both ThermoFisher) were incubated at room temperature for 1 h, after which the slides were washed in PBS. The tissue was then stained with DAPI (Roche, Germany) at room temperature for 10 min, after which it was washed off. The slides were mounted onto coverslips with mowiol (Sigma-Aldrich, UK).

### Statistics

Analysis of two groups was performed using a Mann–Whitney U test. Fishers exact test was used for categorical data (GraphPad Prism v9, GraphPad Software Inc., San Diego, USA). Associations were assessed using Spearman's correlation with rho and p values presented. Results were considered significant if p < 0.05.

## Results

### Subject demographics

This study included 31 COPD subjects and 20 HV-ES as the most relevant control group; characteristics are summarised in Table 1. No significant differences were seen in age, sex, or BMI. Expected differences were seen in lung function.

**Table 1:** Subject demographics

	HV-ES	COPD	P Value
N of subjects	20	31	–
M/F	11/9	25/6	0.0645
Age	67.5 (64.25–72.50)	70.0 (66.0–76.0)	0.2316
FEV1%	100.5 (94.25–109.5)	73.0 (61.0–83.0)	** < 0.0001**
FEV1/FVC ratio %	77.5 (74.25- 79.75)	58.0 (51.0–66.0)	** < 0.0001**
Frequent Exacerbators % (n)	–	45% (14)	–
ICS use % (n)	–	61% (19)	–
ICS (BDP equivalent, µg)*	0 (0.0–0.0)	480 (0–1000)	** < 0.0001**
BMI, kg/m^2^	27.69 (25.65–30.61)	28.49 (26.06–32.15)	0.2863
Blood eosinophils (10^9^/L)	0.1 (0.1–0.25)	0.3 (0.1–0.3)	**0.0264**
BAL eosinophils (%)*	0.6 (0.1–1.0)	0.93 (0.43–2.50)	0.0547

Bold values indicate statistical significance

*BAL* = Bronchoalveolar lavage, *BDP* = beclomethasone dipropionate, *BMI* = body mass index, *COPD* = chronic obstructive pulmonary disease, *FEV1* = forced expiratory volume in one second, *FVC* = forced vital capacity, *HV-ES* = health volunteer ex-smoker who had stopped smoking for at least 6 months, *ICS* = inhaled corticosteroid. Data are presented as median and IQR (interquartile range) unless otherwise indicated. Continuous data were analysed using a one-tailed Mann Whitney test; categorical data were analysed using a Fisher’s Exact test. *Data shown represents 20 HV-ES and 30 COPD subjects

### Eosinophils and associated proteins are increased in COPD

**Fig. 2:**
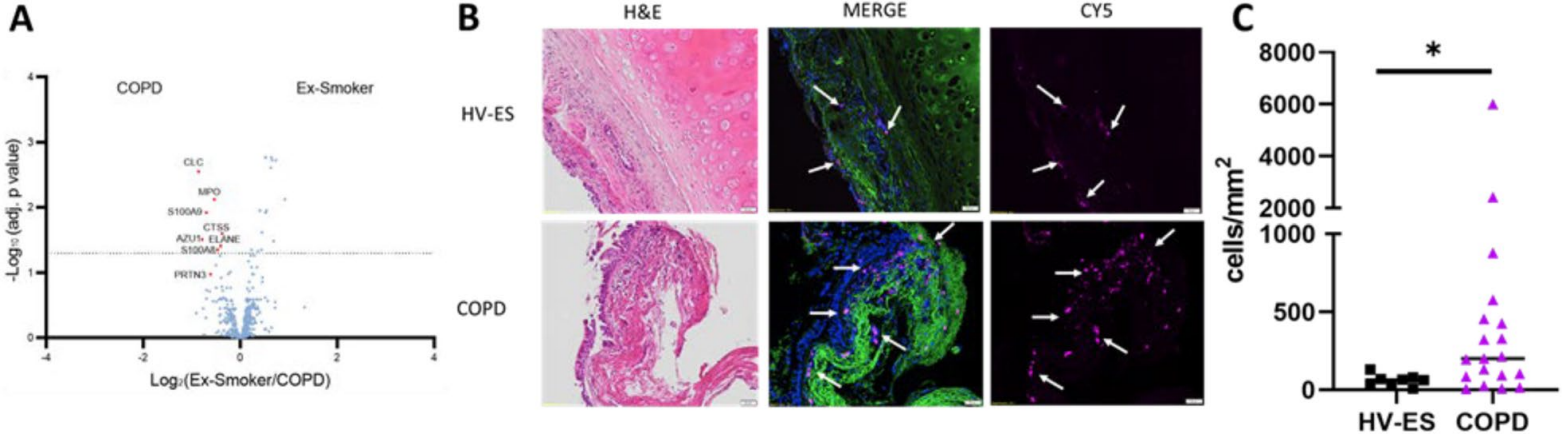
Presence of eosinophils in the lung of HV-ES and COPD subjects. **A** Volcano plot of BAL proteomics in HV-ES vs. COPD. **B** Bright-field Mayer's haematoxylin and eosin (H&E) and immunofluorescence (IF) staining for eosinophils on human lung tissue from HV-ES and COPD subjects. Images were captured using a 20 × objective on an Olympus VS110 slide scanning microscope, scale bar 50 μm. **C** Scatter diagram of eosinophils in the lung tissue of HV-ES and COPD subjects, quantified in cells/mm^2^. Statistical analysis was performed with one-tailed Mann–Whitney’s; p < 0.05*

We first characterised whether blood eosinophil levels were different between COPD subjects and HV-ES and observed a significantly higher number of eosinophils in the blood of COPD subjects (p = 0.0264). Furthermore, we found a trend towards increased proportions of eosinophils in the BAL of COPD subjects vs HV-ES (p = 0.0547) (Table 1). We subsequently conducted an unbiased proteomic analysis of the BAL supernatant from our cohort and, of the 906 proteins detected, 7 proteins were significantly more highly expressed in the BAL of COPD subjects compared to HV-ES (Fig. 2A). These 7 proteins all associated with granulocyte activation, and included proteases such as MPO and ELANE. The protein with the greatest fold-change (Log_2_FC 1.81) and significance (adjp = 0.002) was the eosinophil-associated Charcot-Leyden Crystal (CLC/Galectin-10).

In addition to numbers of eosinophils in blood and BAL, we characterised the presence of tissue eosinophils in formalin-fixed, paraffin-embedded (FFPE) bronchial biopsies from a sub-cohort of 19 COPD subjects and 8 HV-ES (demographics in Table S1). Using immunofluorescence (IF) targeting eosinophil cationic protein (ECP), we identified the presence of tissue eosinophils in both COPD and HV-ES subjects (Fig. 2B). Furthermore, we found significantly greater numbers of eosinophils in tissue from COPD subjects vs HV-ES (p = 0.0108, Fig. 2C). Within this IF subcohort, numbers of blood and BAL eosinophil proportions were also significantly elevated in COPD patients compared with HV-ES (Table S1). Furthermore, the signal of significantly greater CLC/Galectin-10 expression in the COPD vs HV-ES BAL proteome was maintained (Log_2_FC 1.12, adjp = 0.014).

### BAL and tissue eosinophil levels do not correlate with blood eosinophils in COPD

Following demonstration of increased numbers of blood and tissue eosinophils and proportions of BAL eosinophils in COPD vs HV-ES, we next investigated whether levels of eosinophils correlated between each compartment. Within the whole cohort, we found only a weak positive correlation between blood eosinophil numbers and the proportion of eosinophils in BAL (rho = 0.2608, p = 0.0337). There was no correlation between blood eosinophil numbers and the proportion of eosinophils in BAL in COPD subjects alone (rho = 0.01762, p = 0.4632). We found no correlation between blood eosinophil numbers and tissue eosinophil numbers in COPD and HV-ES (rho = 0.1192, p = 0.2768) or in COPD subjects alone in the IF subcohort (rho = −0.09192, p = 0.3541). There was a weak negative correlation between tissue eosinophil numbers and the proportion of eosinophils in BAL in the IF subcohort (rho = −0.3433, p = 0.0430). This was also seen in IF subcohort COPD subjects alone (rho = −0.4961, p = 0.0181).

### Gene expression changes associated with blood eosinophils within COPD

To further investigate differences that may be driving mechanisms underlying eosinophilic inflammation specifically in COPD, we compared COPD subjects who had ≥ 300 cells/µl blood eosinophils or not upon recruitment [4] (Table 2); these subjects are referred to as high and low blood eosinophil COPD subjects respectively. High blood eosinophil COPD subjects had better preserved lung function than low blood eosinophil COPD subjects. However, there were no other significant differences between the groups besides blood eosinophil numbers.

**Table 2:** – COPD subject demographics based on blood eosinophilia

	< 300 cells/µl	≥ 300 cells/µl	P Value
N of subjects	20	11	–
M/F	16/4	9/2	0.6462
Age	70.0 (61.25–75.75)	72.0 (67.0–76.0)	0.1761
FEV1%	69.5 (58.75–79.25)	82.0 (62.0–87.0)	**0.0442**
FEV1/FVC ratio %	57.0 (46.75–62.50)	63.0 (52.0–70.0)	**0.0402**
Pack-years of smoking	47.0 (25.31–59.06)	40 (20.0–60.0)	0.4396
Frequent Exacerbators % (n)	45.0% (9)	45.45% (5)	0.6361
ICS use % (n)	60.0% (12)	63.6% (7)	0.5769
ICS (BDP equivalent, µg)#	480 (0–1000)	730 (0–1250)	0.3782
BMI, kg/m^2^	28.32 (26.07–31.90)	29.88 (24.44–32.27)	0.2641
Blood eosinophils (10^9^/L)	0.1 (0.1–0.2)	0.3 (0.3–0.4)	** < 0.0001**
BAL eosinophils (%)*	1.0 (0.6–3.1)	0.6 (0.2–2.0)	0.2938

Bold values indicate statistical significance

*BAL* = Bronchoalveolar lavage, *BDP* = beclomethasone dipropionate, *BMI* = body mass index, *COPD* = chronic obstructive pulmonary disease, *FEV1* = forced expiratory volume in one second, *FVC* = forced vital capacity, *ICS* = inhaled corticosteroid. Data are presented as median and IQR (interquartile range) unless otherwise indicated. Continuous data were analysed using a one-tailed Mann Whitney test; categorical data were analysed using a Fisher’s Exact test. #ICS dose data shown represents 20 COPD subjects with < 300 cells/µl and 11 COPD subjects with ≥ 300 cells/µl.*BAL data shown represents 19 COPD subjects with < 300 cells/µl and 11 COPD subjects with ≥ 300 cells/µl

**Fig. 3:**
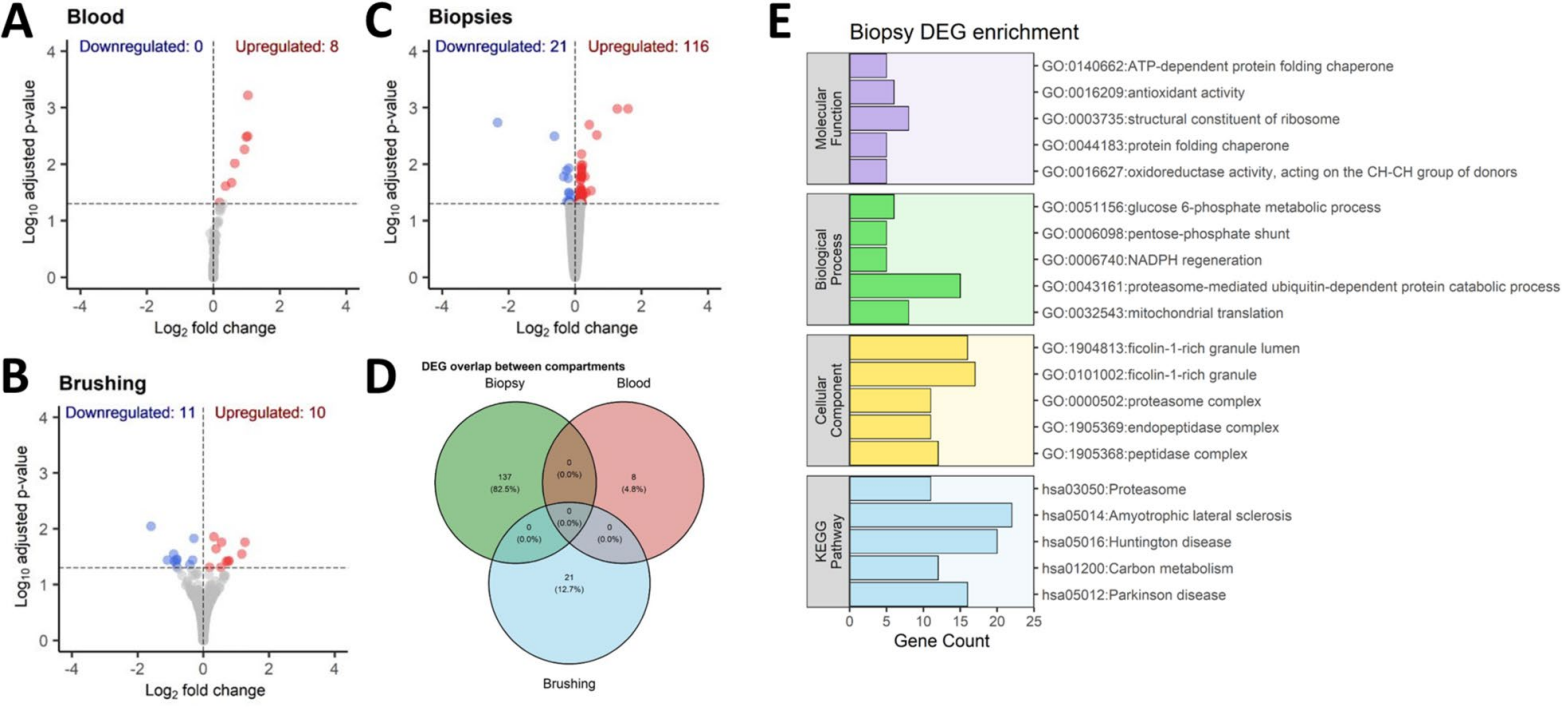
Transcriptomic differences across different compartments of COPD subjects associated with blood eosinophil levels. Differential gene expression analysis compared COPD subjects with high and low blood eosinophils and identified (**A**) 8 DEGs in blood, (**B**) 21 DEGs in epithelial brushings and (**C**) 137 DEGs in bronchial biopsies, but no DEGS (**D**) were shared between sample compartments. **E** Enrichment analysis of DEGs derived from bronchial biopsy samples identified significant enrichment metabolic processes. Gene list enrichment using over representation analysis was performed using clusterProfiler. In (**E**) only the top 5 significantly enriched (adjusted p-value < 0.05) terms and/or pathways are visualised and are ordered by enrichment significance

To understand the processes that might be contributing to increased eosinophils in COPD, we compared genes that are differentially regulated between high and low blood eosinophil COPD subjects in blood, epithelial brushing and bronchial biopsy samples. In blood, 8 differentially expressed genes (DEGs) were identified (Fig. 3A, all upregulated), including *IL5RA, SIGLEC8* and *CLC* (Table S2A). In epithelial brushings, 21 DEGs (Fig. 3B, 10 upregulated, 11 downregulated) were identified (Table S2B). Furthermore, there were 137 DEGs in bronchial biopsies (Fig. 3c, 116 upregulated, 21 downregulated) (Table S2C). There was no overlap of DEGs between the three compartments sampled. (Fig. 3D). Over-representation analysis revealed the bronchial biopsy DEGs were enriched in metabolic processes (Fig. 3E).

### Gene expression changes associated with tissue eosinophils within COPD IF subcohort

To understand which genes may determine an increase in tissue eosinophils, we next investigated both the phenotypic and gene expression associations with tissue eosinophils in COPD subjects within the IF subcohort. As there are no definitive thresholds of high tissue eosinophils in the literature we used the median value of 200 cells/mm^2^ as a threshold to define the low and high tissue eosinophil groups. Unlike the previous blood eosinophil analysis, there was no significant difference in lung function between the 2 groups of COPD subjects (Table 3). In line with the negative correlation described above, the proportion of BAL eosinophils was significantly lower in the tissue eosinophil high group than in the tissue eosinophil low group (Table 3).

**Table 3:** – Immunofluorescence (IF) subcohort COPD subject demographics based on tissue eosinophilia

	< 200 cells/mm^2^l	≥ 200 cells/mm^2^	P Value
N of subjects	9	10	–
M/F	9/0	8/2	0.2632
Age	71.0 (66.5–74.5)	69.5 (64.0–75.0)	0.3077
FEV1%	75.0 (69.5–90.0)	78.5 (61.75–82.25)	0.2170
FEV1/FVC ratio	61.0 (54.5–68.5)	60.0 (49.00–63.75)	0.1934
Pack-years of smoking	60.0 (20.00–71.25)	45.5 (15.75–65.00)	0.3094
Frequent Exacerbators % (n)	44.44% (4)	50.0% (5)	0.5859
ICS use % (n)	66.7% (6)	50.0% (5)	0.3950
ICS (BDP equivalent, µg)#	480 (0–1000)	500 (0–1000)	0.4590
BMI, kg/m^2^	31.44 (28.84–33.09)	24.87 (24.05–32.04)	**0.0380**
Blood eosinophils (10^9^/L)	0.30 (0.15–0.30)	0.25 (0.10–0.48)	0.4708
BAL eosinophils (%)*	1.30 (0.60–5.08)	0.50 (0.20–1.00)	**0.0481**
Tissue eosinophils (cells/mm2)	85.0 (12.0–118.5)	441.0 (295.8–1266.0)	** < 0.0001**

Bold values indicate statistical significance

*BAL* = Bronchoalveolar lavage, *BDP* = beclomethasone dipropionate, *BMI* = body mass index, *COPD* = chronic obstructive pulmonary disease, *FEV1* = forced expiratory volume in one second, *FVC* = forced vital capacity, *ICS* = inhaled corticosteroid. Data are presented as median and IQR (interquartile range) unless otherwise indicated. Continuous data were analysed using a one-tailed Mann Whitney test; categorical data were analysed using a Fisher’s Exact test. #ICS dose data shown represents 8 COPD subjects with < 300 cells/µl and 10 COPD subjects with ≥ 300 cells/µl.*BAL data shown represents 9 COPD subjects with < 200 cells/mm^2^ and 9 COPD subjects with ≥ 200 cells/mm^2^

**Fig. 4:**
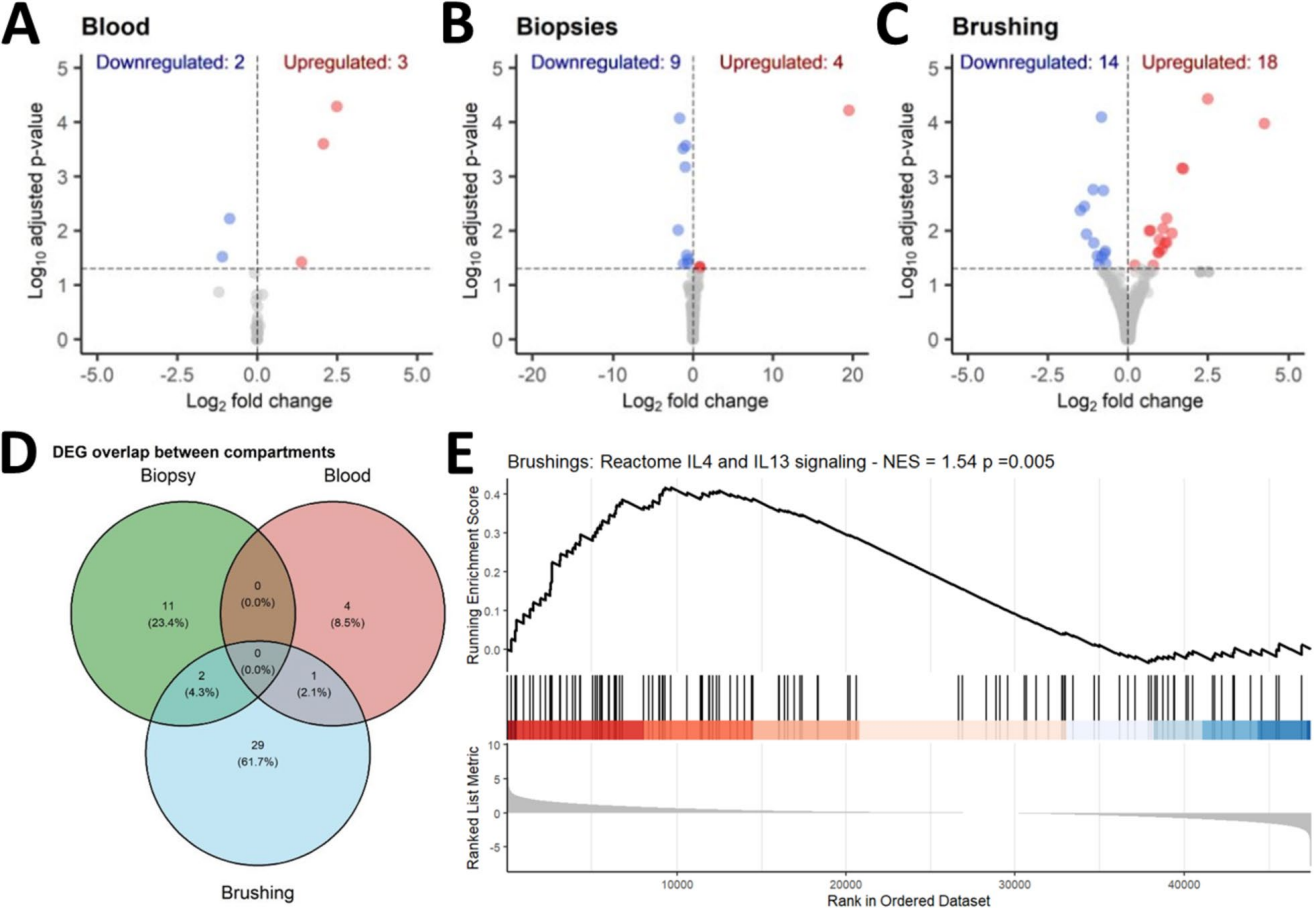
Transcriptomic differences across different compartments of COPD subjects associated with tissue eosinophil levels Differential gene expression analysis on the immunofluorescence (IF) sub cohort compared COPD subjects with high and low tissue eosinophil levels and found (**A**) 5 DEGs in blood, (**B**) 13 DEGs in bronchial biopsies and (**C**) 32 DEGs in epithelial brushings, and very few DEGS (**D**) were shared between sample compartments. (**E**) Gene set enrichment analysis (GSEA) on the IF subcohort ranked epithelial brushings gene list identified positive significant enrichment of the REACTOME IL-4/IL-13 pathway (R-HSA-6785807). GSEA was performed using clusterProfiler. NES = normalised enrichment score.

Analysing gene expression differences between COPD subjects with high and low tissue eosinophils from this smaller IF subcohort revealed 5 DEGs in blood (Fig. 4A, 3 upregulated, 2 downregulated). In epithelial brushings, 32 DEGs (18 upregulated, 14 downregulated) were identified, including *POSTN*, encoding a matrix protein induced by IL-13 (Fig. 4B) [41]. In bronchial biospies, 13 DEGs (4 upregulated, 9 downregulated) were identified (Fig. 4C). Again, no DEGs were commonly differentially expressed between all 3 compartments (Fig. 4D). However, *IFI6* and *LRRC37A* were downregulated in both epithelial brushings and biopsies (Table S3). Gene set enrichment anlaysis (GSEA) was performed to further investigate the potential impact of IL-13 signalling in epithelial brushings and identified positive significant enrichment of the REACTOME IL-13/IL-4 signaling pathway (NES = 1.54, p = 0.005, Fig. 4E).

### Tissue IL-5Rα expression

**Fig. 5:**
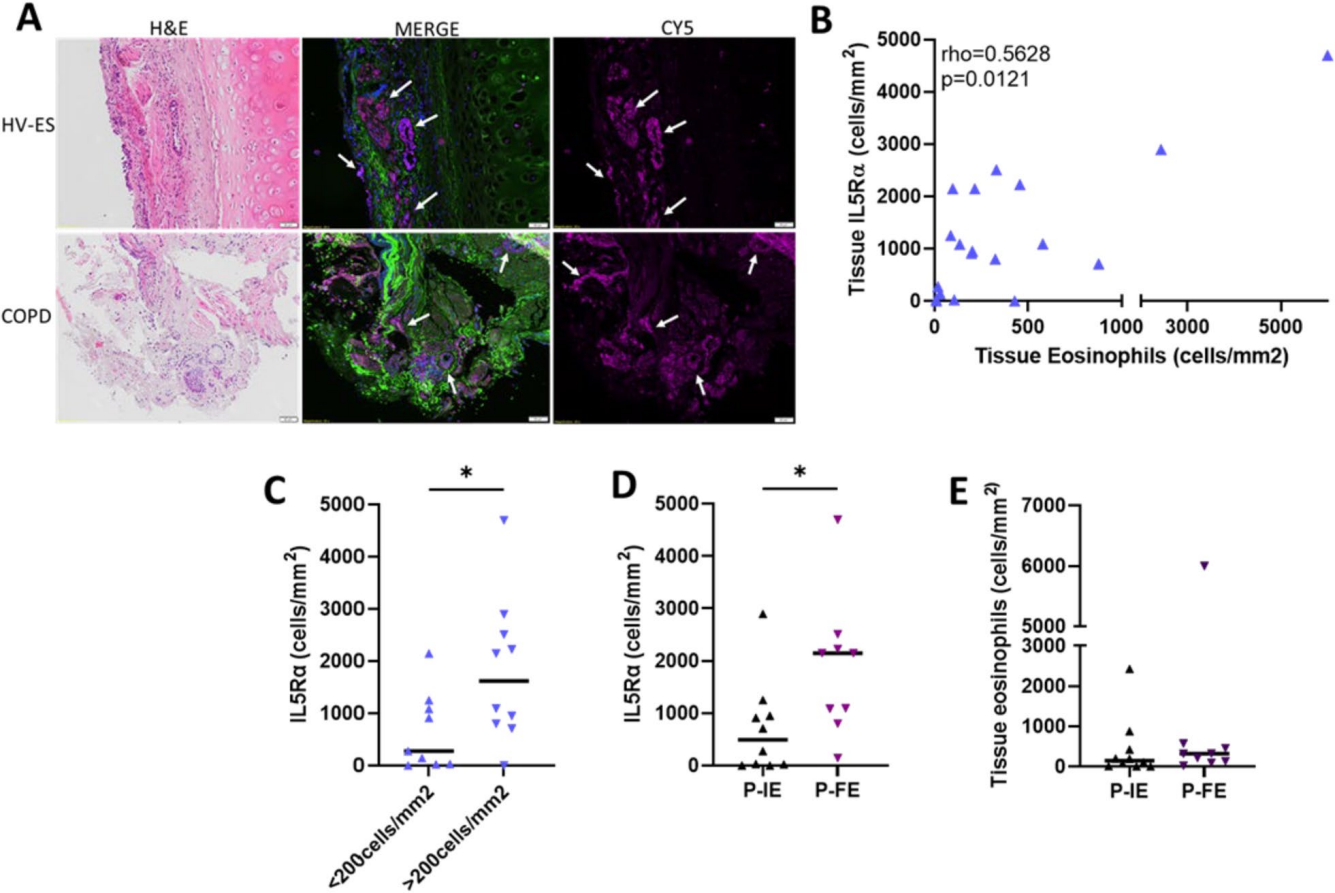
Tissue IL5Rα expression and associations with tissue eosinophils. **A** Bright-field Mayer's haematoxylin and eosin (H&E) and IF staining for IL5Rα on human lung tissue from HV-ES and COPD subjects. Images were captured using a 20 × objective on an Olympus VS110 slide scanning microscope, scale bar 50 μm. **B** Spearman’s non-parametric correlation between eosinophils and IL5Rα in the lung tissue of COPD subjects. **C** IL5Rα in the lung tissue of COPD subjects with tissue eosinophil count below 200 cell/mm^2^ and above 200 cells/mm^2^. **D** Distribution of IL5Rα in lung tissue of COPD subjects separated into infrequent (P-IE) and frequent exacerbators (P-FE) phenotype. **E** Eosinophils in the lung tissue of COPD subjects with P-IE and P-FE phenotype. Two-tailed Mann–Whitney’s statistical analysis was performed; p < 0.05*

To further investigate why tissue eosinophils may be increased in COPD, we also probed the biopsy tissue in the IF subcohort for the α-subunit of the receptor for the eosinophil survival factor IL-5 (IL5Rα). IL5Rα appeared to be widely expressed in submucosal glandular tissue in addition to eosinophils (Fig. 5A). In contrast to tissue eosinophils, there was no significant difference in total expression of IL5Rα between COPD subjects and HV-ES subjects in the IF subcohort (p = 0.1633). There was also no significant differential expression of IL5Rα between COPD patients with high blood eosinophils compared to COPD patients with low blood eosinophils in the IF subcohort (p = 0.2294).

In the IF subcohort, we observed a weak but significant correlation between tissue IL5Rα expression and tissue eosinophil numbers in COPD and HV-ES (rho = 0.3148, p = 0.0478) and this correlation strengthened when only COPD subjects from the IF subcohort were included (rho = 0.5628, p = 0.0121, Fig. 5B). There was significant differential expression of IL5Rα between COPD high tissue eosinophils vs COPD low tissue eosinophils (p = 0.031, Fig. 5C).

In terms of IF subcohort patient phenotype, there was a significant increase in tissue IL5Rα expression in those patients who experienced frequent exacerbations (p = 0.0131, Fig. 5D) but tissue eosinophils were not different based on exacerbation history (p = 0.1388, Fig. 5E).

## Discussion

Our study used a deeply-phenotyped cohort to demonstrate a clear increase in eosinophils in the blood, BAL and tissue of mild-moderate COPD patients compared to healthy, ex-smokers. These eosinophils appear to be activated in the lung due to increased levels of CLC/Galectin-10 in the BAL of COPD patients. We further found that blood eosinophil levels did not correlate with eosinophil levels in BAL or lung tissue in COPD subjects, raising new questions about the utility of this measure alone for understanding lung tissue inflammation. We observed a significant correlation between tissue eosinophils and tissue IL5Rα expression. However, lung IL5Rα expression was not limited to eosinophils, with substantial IL5Rα expression in submucosal glands, further indicating a possible role for IL-5 receptor signalling in the epithelium [42]. Of note, in the context of recent trials, tissue eosinophils were associated with DEGs known to be regulated by IL-13.

Our study highlights the complexity of eosinophilic inflammation in COPD with the impact of eosinophils on disease characteristics being subtle and related to the compartment in which eosinophils are measured. Current GOLD guidelines recommend using blood eosinophil counts ≥ 300 cells/µl as a biomarker to identify those with the greatest likelihood of treatment benefit with inhaled corticosteroids [4]. Using RNASeq, bronchial biopsies had the greatest number of DEGs that associated with this measure of increased blood eosinophils with enrichment of genes in numerous metabolic pathways. However, there were very few bronchial biopsy genes associated with tissue eosinophilia. These data highlight the impact that measuring eosinophils in different compartments has on defining patient lung relevant endotypes. Furthermore, they suggest a complex relationship between raised eosinophils and T2-gene signatures, independent of IL-5, which has implications for using eosinophils as a treatable trait in COPD.

Our data also highlight the complexity in defining raised eosinophils in COPD as whilst previous data supports a correlation between blood and sputum [13, 14, 43], blood does not appear to be a good biomarker of tissue or BAL eosinophils in patients and we observed marked compartmental differences. Adding further complexity, there was a negative correlation between BAL and tissue eosinophils. Eltoboli et al. (2015) previously demonstrated an association of increased tissue eosinophils with reticular basement membrane thickening of COPD patients, suggesting that the presence of these cells in tissue is important for disease [12]. Whilst there is some evidence for the stability of eosinophil numbers in bronchial biopsies from COPD patients [11, 44], our observation of a lack of correlation between compartments agrees with a study including 294 COPD patients that also demonstrated no correlation between compartments [16]. The weak correlations between blood and BAL suggests vascular leakage may be playing a role in the detection of eosinophils in the BAL but not residency of these cells in the tissue. Indeed, as there is a negative correlation between the tissue and the airway lumen, this observation might suggest that opposing mechanisms are involved in retaining eosinophils in the tissue or trafficking to the lumen.

Despite the lack of correlation between blood and tissue eosinophils, we did observe a gene expression signature associated with blood eosinophils in the bronchial biopsy tissue, many of which were associated with metabolic processes. This increased expression of metabolic genes could be a result of the ongoing energy demands of eosinophil-driven inflammatory processes in the tissue [45]. Alternatively, given that high blood eosinophils were associated with more preserved lung function, this increase in metabolic processes may represent ongoing repair processes [46]. Further work will be required to either confirm or refute these speculations.

To understand the drivers of increased tissue eosinophils in COPD, we investigated the gene expression differences associated with this trait. In brushings, there is further evidence of increased T2 inflammatory processes, with increased expression of *POSTN*, encoding periostin. Periostin expression in the epithelium is increased by the T2-cytokine, IL-13, and in asthma has been associated with both increased airway eosinophils and mucus secretion [41]. In biopsies, there was also an increase in *CCL26*, encoding the eosinophil chemokine eotaxin-3, expression of which is also known to be increased by IL-4 and IL-13 [47]. Our GSEA further supports the presence of T2 inflammation through identification of positive enrichment of IL-13/IL-4 signalling in the brushings dataset. This evidence of increased IL-13 signalling in patients with increased tissue eosinophils may provide some explanation for the observed efficacy of dupliumab in COPD [5].

Taken together, these data suggest that elevated eosinophil counts are common in COPD but that tissue eosinophils may be more relevant to continuing disease processes than blood or luminal eosinophils. This observation may explain the limited efficacy of anti-IL-5 treatments in preventing COPD exacerbations and disease progression as these studies used blood eosinophils to stratify these patients [48, 49]. These anti-IL-5 treatments are effective at reducing blood eosinophils, but our data suggest that IL-13-driven pathways may be responsible for the maintenance and survival of these cells in COPD lung tissue. Further support for this observation comes from the BOREAS trail which demonstrated no significant effect of 52-week dupliumab treatment on blood eosinophil levels despite a significant effect on exacerbations and prebronchodilator FEV1, although FeNO was reduced [5].

We recognise that this study is not without its limitations. Due to the deep characterisation of subjects and intensive sampling, the cohort is small and the study captured only cross-sectional measures, providing no insight into eosinophil stability in the different compartments over time and disease states (e.g. stable vs exacerbation). We thus cannot fully rule out that lack of correlations are not due to cohort size. In particular, the size of the IF cohort was limited due to capture of tissue for RNASeq being prioritised. Additionally, sputum data was not available from all subjects and thus we have no data as to the correlation between tissue and sputum eosinophils. Our study included mild-moderate COPD patients and gives insights about earlier disease. Comparison of our findings with those in more severe disease in future studies is merited.

Our study demonstrates a clear increase in eosinophils in COPD compared to health in both blood, BAL and lung tissue. Furthermore, we demonstrate that blood eosinophil levels did not correlate with eosinophils nor IL5Rα expression in tissue. We have identified differentially expressed genes that associated with eosinophils in different compartments. Blood eosinophils do define an inflammatory endotype that can be detected in lung tissue but do not reflect the expected IL-5-mediated pathways. Further delineating the complex signalling pathways driving tissue eosinophilic inflammation in COPD with mechanistic studies could provide information on optimal targeting of existing and novel therapies.

## Take home message

There is active eosinophilic inflammation in the lungs of COPD patients compared to controls but blood eosinophils alone do not reflect tissue eosinophils or gene expression. Understanding of lung eosinophil biology is needed to tailor new therapy.

## Supplementary Information

Additional file 1

Additional file 2

Additional file 3

## Data Availability

The datasets generated and analysed during the current study are not publicly available in order to protect the privacy of all individuals whose data we have collected, stored, and analysed. However, data may be made available upon reasonable request by applying through the established Data Request Portal through which Researchers can request access to de-identified clinical data (https://vivli.org), after which, clinical data may be made available upon review of the patient consent forms, scientific merit of the proposal, and signature of a data sharing/collaboration agreement. This mechanism allows controlled, risk-managed accessibility of the data and at the same time safeguards patients’ confidentiality.

## Abbreviations

BAL: Bronchoalveolar lavage
CLC: Charcot-Leyden Crystal
COPD: Chronic Obstructive Pulmonary Disease
DEGs: Differentially-expressed genes
FC: Log2-fold change
FeNO: Fractional exhaled nitric oxide
GOLD: Global Initiative for Obstructive Lung Disease
GSEA: Geneset enrichment analysis
HV-ES: Control ex-smokers
HV-NS: Control never-smokers
ICS: Inhaled corticosteroids
IF: Immunofluorescence
IL: Interleukin
MS: Mass spectrometry
P-IE: COPD infrequent exacerbators
P-FE: COPD frequent exacerbators
RNASeq: Ribonucleic acid sequencing

## References

[1] Rabe KF, Watz H. Chronic obstructive pulmonary disease. Lancet. 2017;389(10082):1931–40. 28513453 10.1016/S0140-6736(17)31222-9

[2] Tashkin DP, Wechsler ME. Role of eosinophils in airway inflammation of chronic obstructive pulmonary disease. Int J Chron Obstruct Pulmon Dis. 2018;13:335–49. 29403271 10.2147/COPD.S152291 PMC5777380

[3] Ho J, He W, Chan MTV, Tse G, Liu T, Wong SH, et al. Eosinophilia and clinical outcome of chronic obstructive pulmonary disease: a meta-analysis. Sci Rep. 2017;7(1):13451. 29044160 10.1038/s41598-017-13745-x PMC5647332

[4] Global Initiative for Chronic Obstructive Lung Disease. Global Strategy for the Diagnosis, Management, and Prevention of Chronic Obstructive Pulmonary Disease - 2023 report. www.goldcopd.org Accessed 14 Jan 2023

[5] Bhatt Surya P, Rabe Klaus F, Hanania Nicola A, Vogelmeier Claus F, Cole J, Bafadhel M, et al. Dupilumab for COPD with Type 2 inflammation indicated by eosinophil counts. N Engl J Med. 2023;389(3):205–14. 37272521 10.1056/NEJMoa2303951

[6] Rutgers S, Timens W, Kaufmann H, van der Mark T, Koeter G, Postma D. Comparison of induced sputum with bronchial wash, bronchoalveolar lavage and bronchial biopsies in COPD. Eur Respir J. 2000;15(1):109–15. 10678630 10.1183/09031936.00.15110900

[7] Saha S, Brightling CE. Eosinophilic airway inflammation in COPD. Int J Chron Obstruct Pulmon Dis. 2006;1(1):39–47. 18046901 10.2147/copd.2006.1.1.39 PMC2706606

[8] Brightling CE, Monteiro W, Ward R, Parker D, Morgan MD, Wardlaw AJ, Pavord ID. Sputum eosinophilia and short-term response to prednisolone in chronic obstructive pulmonary disease: a randomised controlled trial. Lancet. 2000;356(9240):1480–5. 11081531 10.1016/S0140-6736(00)02872-5

[9] Kolsum U, Donaldson GC, Singh R, Barker BL, Gupta V, George L, et al. Blood and sputum eosinophils in COPD; relationship with bacterial load. Respir Res. 2017;18(1):88. 28482840 10.1186/s12931-017-0570-5 PMC5422866

[10] Park HY, Chang Y, Kang D, Hong YS, Zhao D, Ahn J, et al. Blood eosinophil counts and the development of obstructive lung disease: the Kangbuk Samsung health study. Eur Respir J. 2021. 10.1183/13993003.03823-2020. 33737406 10.1183/13993003.03823-2020

[11] Kolsum U, Damera G, Pham TH, Southworth T, Mason S, Karur P, et al. Pulmonary inflammation in patients with chronic obstructive pulmonary disease with higher blood eosinophil counts. J Allergy Clin Immunol. 2017;140(4):1181-4.e7. 28506852 10.1016/j.jaci.2017.04.027

[12] Eltboli O, Mistry V, Barker B, Brightling CE. Relationship between blood and bronchial submucosal eosinophilia and reticular basement membrane thickening in chronic obstructive pulmonary disease. Respirology. 2015;20(4):667–70. 25645275 10.1111/resp.12475 PMC4833195

[13] Bafadhel M, McKenna S, Terry S, Mistry V, Reid C, Haldar P, et al. Acute exacerbations of chronic obstructive pulmonary disease. Am J Respir Crit Care Med. 2011;184(6):662–71. 21680942 10.1164/rccm.201104-0597OC

[14] Kim VL, Coombs NA, Staples KJ, Ostridge KK, Williams NP, Wootton SA, et al. Impact and associations of eosinophilic inflammation in COPD: analysis of the AERIS cohort. Eur Respir J. 2017;50(4):1700853. 29025891 10.1183/13993003.00853-2017

[15] Barber C, Lau L, Ward JA, Daniels T, Watson A, Staples KJ, et al. Sputum processing by mechanical dissociation: a rapid alternative to traditional sputum assessment approaches. Clin Respir J. 2021;15(7):800–7. 33749082 10.1111/crj.13365

[16] Turato G, Semenzato U, Bazzan E, Biondini D, Tinè M, Torrecilla N, et al. Blood eosinophilia neither reflects tissue eosinophils nor worsens clinical outcomes in chronic obstructive pulmonary disease. Am J Respir Crit Care Med. 2018;197(9):1216–9. 29035097 10.1164/rccm.201708-1684LE

[17] Bakakos A, Rovina N, Bakakos P. Treatment challenges in severe eosinophilic asthma: differential response to Anti-IL-5 and Anti-IL-5R therapy. Int J Mol Sci. 2021. 10.3390/ijms22083969. 33921360 10.3390/ijms22083969 PMC8069413

[18] Day K, Ostridge K, Conway J, Cellura D, Watson A, Spalluto CM, et al. Interrelationships among small airways dysfunction, neutrophilic inflammation, and exacerbation frequency in COPD. Chest. 2021;159(4):1391–9. 33245876 10.1016/j.chest.2020.11.018

[19] Watson A, Spalluto CM, McCrae C, Cellura D, Burke H, Cunoosamy D, et al. Dynamics of IFN-β responses during respiratory viral infection. Insights for therapeutic strategies. Am J Respir Crit Care Med. 2020;201(1):83–94. 31461630 10.1164/rccm.201901-0214OC

[20] Ostridge K, Gove K, Paas KHW, Burke H, Freeman A, Harden S, et al. Using Novel computed tomography analysis to describe the contribution and distribution of emphysema and small airways disease in chronic obstructive pulmonary disease. Ann Am Thorac Soc. 2019;16(8):990–7. 30892055 10.1513/AnnalsATS.201810-669OC

[21] Watson A, Öberg L, Angermann B, Spalluto CM, Hühn M, Burke H, et al. Dysregulation of COVID-19 related gene expression in the COPD lung. Respir Res. 2021;22(1):1–13. 34051791 10.1186/s12931-021-01755-3 PMC8164067

[22] Hristova VA, Watson A, Chaerkady R, Glover MS, Ackland J, Angermann B, et al. Multiomics links global surfactant dysregulation with airflow obstruction and emphysema in COPD. ERJ Open Res. 2022;9(3):00378-2022. 10.1183/23120541.00378-2022. 37228288 10.1183/23120541.00378-2022 PMC10204810

[23] A Quality Control Tool for High Throughput Sequence Data. 2015. https://www.bioinformatics.babraham.ac.uk/projects/fastqc/ Accessed 5 Jan 2024

[24] Okonechnikov K, Conesa A, García-Alcalde F. Qualimap 2: advanced multi-sample quality control for high-throughput sequencing data. Bioinformatics. 2016;32(2):292–4. 26428292 10.1093/bioinformatics/btv566 PMC4708105

[25] Li H, Handsaker B, Wysoker A, Fennell T, Ruan J, Homer N, et al. The Sequence Alignment/Map format and SAMtools. Bioinformatics. 2009;25(16):2078–9. 19505943 10.1093/bioinformatics/btp352 PMC2723002

[26] Dobin A, Davis CA, Schlesinger F, Drenkow J, Zaleski C, Jha S, et al. STAR: ultrafast universal RNA-seq aligner. Bioinformatics. 2013;29(1):15–21. 23104886 10.1093/bioinformatics/bts635 PMC3530905

[27] Ewels P, Magnusson M, Lundin S, Käller M. MultiQC: summarize analysis results for multiple tools and samples in a single report. Bioinformatics. 2016;32(19):3047–8. 27312411 10.1093/bioinformatics/btw354 PMC5039924

[28] Gaspar JM. NGmerge: merging paired-end reads via novel empirically-derived models of sequencing errors. BMC Bioinform. 2018;19(1):536. 10.1186/s12859-018-2579-2 PMC6302405 30572828

[29] Patro R, Duggal G, Love MI, Irizarry RA, Kingsford C. Salmon provides fast and bias-aware quantification of transcript expression. Nat Methods. 2017;14(4):417–9. 28263959 10.1038/nmeth.4197 PMC5600148

[30] Di Tommaso P, Chatzou M, Floden EW, Barja PP, Palumbo E, Notredame C. Nextflow enables reproducible computational workflows. Nat Biotechnol. 2017;35(4):316–9. 28398311 10.1038/nbt.3820

[31] Grüning B, Dale R, Sjödin A, Chapman BA, Rowe J, Tomkins-Tinch CH, et al. Bioconda: sustainable and comprehensive software distribution for the life sciences. Nat Methods. 2018;15(7):475–6. 29967506 10.1038/s41592-018-0046-7 PMC11070151

[32] Love MI, Huber W, Anders S. Moderated estimation of fold change and dispersion for RNA-seq data with DESeq2. Genome Biol. 2014;15(12):550. 25516281 10.1186/s13059-014-0550-8 PMC4302049

[33] Stephens M. False discovery rates: a new deal. Biostatistics. 2017;18(2):275–94. 27756721 10.1093/biostatistics/kxw041 PMC5379932

[34] R Core Team. R: A language and environment for statistical computing. R Foundation for Statistical Computing, Vienna, Austria. 2019. http://www.R-project.org/ Accessed 1 Sep 2020.

[35] Soneson C, Love MI, Robinson MD. Differential analyses for RNA-seq: transcript-level estimates improve gene-level inferences. F1000Res. 2015;4:1521. 26925227 10.12688/f1000research.7563.1 PMC4712774

[36] Wu T, Hu E, Xu S, Chen M, Guo P, Dai Z, et al. clusterProfiler 4.0: A universal enrichment tool for interpreting omics data. Innovation (Camb). 2021;2(3):100141. 34557778 10.1016/j.xinn.2021.100141 PMC8454663

[37] Carlson M (2023). org.Hs.eg.db: Genome wide annotation for Human. R package version 3.18.0. Accessed 5 Jan 2024

[38] Yu G, He QY. ReactomePA: an R/Bioconductor package for reactome pathway analysis and visualization. Mol Biosyst. 2016;12(2):477–9. 26661513 10.1039/c5mb00663e

[39] Lawson MJ, Katsamenis OL, Chatelet D, Alzetani A, Larkin O, Haig I, et al. Immunofluorescence-guided segmentation of three-dimensional features in micro-computed tomography datasets of human lung tissue. R Soc Open Sci. 2021;8(11): 211067. 34737879 10.1098/rsos.211067 PMC8564621

[40] Hsia CC, Hyde DM, Ochs M, Weibel ER. An official research policy statement of the American thoracic society/European respiratory society: standards for quantitative assessment of lung structure. Am J Respir Crit Care Med. 2010;181(4):394–418. 20130146 10.1164/rccm.200809-1522ST PMC5455840

[41] Burgess JK, Jonker MR, Berg M, ten Hacken NTH, Meyer KB, van den Berge M, et al. Periostin: contributor to abnormal airway epithelial function in asthma? Eur Respir J. 2021;57(2):2001286. 32907887 10.1183/13993003.01286-2020

[42] Barretto KT, Brockman-Schneider RA, Kuipers I, Basnet S, Bochkov YA, Altman MC, et al. Human airway epithelial cells express a functional IL-5 receptor. Allergy. 2020;75(8):2127–30. 32246831 10.1111/all.14297 PMC7387204

[43] Negewo NA, McDonald VM, Baines KJ, Wark PA, Simpson JL, Jones PW, Gibson PG. Peripheral blood eosinophils: a surrogate marker for airway eosinophilia in stable COPD. Int J Chron Obstruct Pulmon Dis. 2016;11:1495–504. 27445469 10.2147/COPD.S100338 PMC4936821

[44] Higham A, Leow-Dyke S, Jackson N, Singh D. Stability of eosinophilic inflammation in COPD bronchial biopsies. Eur Respir J. 2020. 10.1183/13993003.00622-2020. 32471933 10.1183/13993003.00622-2020

[45] Porter L, Toepfner N, Bashant KR, Guck J, Ashcroft M, Farahi N, Chilvers ER. Metabolic profiling of human eosinophils. Front Immunol. 2018;9:1404. 30013547 10.3389/fimmu.2018.01404 PMC6036296

[46] Liu G, Summer R. Cellular metabolism in lung health and disease. Annu Rev Physiol. 2019;81:403–28. 30485759 10.1146/annurev-physiol-020518-114640 PMC6853603

[47] Kagami S, Saeki H, Komine M, Kakinuma T, Tsunemi Y, Nakamura K, et al. Interleukin-4 and interleukin-13 enhance CCL26 production in a human keratinocyte cell line. HaCaT cells Clin Exp Immunol. 2005;141(3):459–66. 16045735 10.1111/j.1365-2249.2005.02875.x PMC1809447

[48] Pavord ID, Chanez P, Criner GJ, Kerstjens HAM, Korn S, Lugogo N, et al. Mepolizumab for eosinophilic chronic obstructive pulmonary disease. N Engl J Med. 2017;377(17):1613–29. 28893134 10.1056/NEJMoa1708208

[49] Criner GJ, Celli BR, Brightling CE, Agusti A, Papi A, Singh D, et al. Benralizumab for the prevention of COPD exacerbations. N Engl J Med. 2019;381(11):1023–34. 31112385 10.1056/NEJMoa1905248

