# Supplementary material for: Lung IL-13 gene signatures are associated with raised tissue eosinophils in COPD
Source: Respir Res. 2025 Mar 25;26:114. doi: 10.1186/s12931-025-03177-x (PMC11938775; doi:10.1186/s12931-025-03177-x)
Supplement: Supplementary file 1 — Additional file 1 [file 12931_2025_3177_MOESM1_ESM.docx]

**Online supplement:**

**Lung IL13 gene signatures are associated with raised tissue eosinophils in COPD**

Karl J Staples^1,2#^, Jodie Ackland^1^, Sruthymol Lukose^1,2^, Bastian Angermann^3^, Graham Belfield^4^, Maria Belvisi^5,6^, Raghothama Chaerkady^7^, Damla Etal^4^, Ashley Heinson^1^, Sonja Hess^7^, Ventzislava A. Hristova^7^, Michael Hühn^3^, Christopher McCrae^8^, Daniel Muthas^3^, Lisa Öberg^3^, Kristoffer Ostridge^1,3^, Adam Platt^9^, C. Mirella Spalluto^1^, Alastair Watson^1^, Tom Wilkinson^1,2^ on behalf of the MICAII study group^&^

# MICAII Study Group

Bastian Angerman ^1^

Stephanie Ashenden ^2^

Sarah Bawden ^3^

Graham Belfield ^2^

Maria G. Belvisi ^1,4^

Aurelie Bornot ^2^

Jerome Bouquet ^5^

Hannah Burke ^3, 6^

Carolina Caceres ^5^

Raghothama Chaerkady ^7^

Doriana Cellura ^3, 6^

Chia-Chien Chiang ^8^

Kerry Day ^3, 6^

Antonio DiGiandomenico ^5^

Hanna Duàn ^1^

Ulrika Edvardsson ^9^

Damla Etal ^2^

Anna Freeman ^3, 6^

Matthew S. Glover ^7^

Vancheswaran Gopalakrishnan ^5^

Stephen Harden ^10^

Sonja Hess ^7^

Alex Hicks ^3, 6^

Ventzislava A. Hristova ^7^

Michael Hühn ^1^

Fredrik Karlsson ^2^

Shameer Khader ^8^

Glenda Lassi ^1^

Alex Mackay ^1,4^

Christopher McCrae ^1^

Christopher Morehouse ^5^

Daniel Muthas ^1^

Karl Nordström ^2^

Steven Novick ^2^

Esther Nyimbili ^3^

Kristoffer Ostridge ^1, 6^

Lisa Öberg ^1^

Adam Platt ^14^

Laura Presland ^3^

Xiaotao Qu ^8^

Nicola Rayner ^3^

Pedro Rodrigues ^3^

Bret Sellman ^5^

Gary Sims ^1^

Cosma Mirella Spalluto ^6^

Andria Staniford ^3^

Karl J. Staples ^3, 6^

Bruce Thompson ^12^

Outi Vaarala^13^

Junmin Wang ^7^

Paul Warrener ^5^

Alastair Watson ^6^

Nicholas P. Williams ^3, 6^

Tom M. A Wilkinson ^3, 6^

Wen Yu ^8^

Bairu Zhang ^2^

Tianhui Zhang ^2^

Natalie van Zuydam ^2^

^1^ Research and Early Development, Respiratory & Immunology, BioPharmaceuticals R&D, AstraZeneca, Gothenburg, Sweden

^2^ Translational Genomics, Discovery Biology, Discovery Sciences, BioPharmaceuticals R&D, AstraZeneca, Gothenburg, Sweden

^3^ NIHR Southampton Biomedical Research Centre, Southampton, UK

^4^ National Heart & Lung Institute, Imperial College London, London, UK

^5^ Microbial Sciences, BioPharmaceuticals R&D, AstraZeneca, Gothenburg, Sweden

^6^ Faculty of Medicine, University of Southampton, Southampton, UK

^7^ Dynamic Omics, Centre for Genomics Research, Discovery Sciences, BioPharmaceuticals R&D, AstraZeneca, Gaithersburg, USA

^8^ Data Science and Artificial Intelligence, BioPharmaceuticals R&D, AstraZeneca, Gothenburg, Sweden

^9^ Business Development and Licensing, BioPharmaceuticals R&D, AstraZeneca, Gothenburg, Sweden

^10^ University Hospital Southampton NHS Foundation Trust, Southampton, UK

^11^ Research and Early Development, Respiratory & Immunology, BioPharmaceuticals R&D, AstraZeneca, Gaithersburg, USA

^12^ Swinburne University of Technology Melbourne, Australia

^13^ Faculty of Medicine, University of Helsinki, Helsinki, Finland

^14^ Research and Early Development, Respiratory & Immunology, BioPharmaceuticals R&D, AstraZeneca, Cambridge, UK

Table S1 – IF subcohort demographics

|  | HV-ES | COPD | P Value |
| --- | --- | --- | --- |
| N of patients | 8 | 19 | - |
| Sex (M/F) | 6/2 | 17/2 | 0.5583 |
| Age | 67.5 (64.25-71.75) | 71.0 (66.0-75.0) | 0.1901 |
| FEV1% predicted | 100.5 (86.75-109.3) | 77.0 (68.0-83.0) | **0.0001** |
| FEV1/FVC ratio | 80, IQR= 4 | 77.5 IQR= 4.5 | **<0.0001** |
| Pack-years of smoking | 26 (21.25-40.0) | 51 (20.0-67.5) | 0.2274 |
| Frequent Exacerbators % (n) | .- | 47.4% (9) | - |
| ICS use % (n) | - | 57.9% (11) | - |
| ICS (BDP equivalent, µg)* | 0 (0.0-0.0) | 480 (0-1000) | **0.0082** |
| BMI, kg/m^2^ | 28.5 (26.63-32.11) | 29.88 (24.44-32.20) | 0.4435 |
| Blood eosinophils (10^9^/L) | 0.1 (0.1-0.25) | 0.3 (0.1-0.3) | **0.0480** |
| BAL eosinophils (%)* | 0.1 (0.0- 0.50) | 0.6 (0.2-2.5) | **0.0154** |

*BAL = Bronchoalveolar lavage, BMI = body mass index, COPD = chronic obstructive pulmonary disease, FEV 1 = forced expiratory volume in one second, FVC = forced vital capacity,* *HV-ES = health volunteer ex-smoker who had stopped smoking for at least 6 months Data are presented as median and IQR (interquartile range) unless otherwise indicated. Continuous data were analysed using a one-tailed Mann Whitney test; categorical data were analysed using a Fisher’s Exact test. #ICS dose data shown represents 8 HV-ES and 18 COPD subjects *BAL data shown represents 8 HV-ES and 16 COPD subjects.*

Table S2A, S2B & S2C in attached Excel file.

Table S3A, S3B & S3C in attached Excel file

Table S4 – COPD blood eosinophil groups in the IF subcohort

|  | <300 cells/µl | ≥300 cells/µl | P Value |
| --- | --- | --- | --- |
| N of patients | 9 | 10 | - |
| Sex (M/F) | 9/0 | 8/2 | 0.2632 |
| Age | 70.0 (66.0-73.5) | 72.0 (66.75-75.25) | 0.2176 |
| FEV1% predicted | 73.0 (68.0-81.5) | 82.0 (61.25-86.75) | 0.2818 |
| FEV1/FVC ratio | 59.0 (56.0-63.0) | 63.5 (51.75-70.25) | 0.2423 |
| Pack-years of smoking | 56.0 (15.0-73.75) | 45.5 (20.0-63.75) | 0.4117 |
| Frequent Exacerbators % (n) | 55.56% (5) | 40.0% (4) | 0.4141 |
| ICS use % (n) | 55.56% (5) | 60.0% (6) | 0.6050 |
| ICS (BDP equivalent, µg)* | 460 (0-1000) | 1000 (0-1500) | 0.2702 |
| BMI, kg/m^2^ | 30.71 (24.86-32.98) | 29.32 (24.44-32.22) | 0.4299 |
| Blood eosinophils (10^9^/L) | 0.1 (0.1-0.2) | 0.3 (0.30-0.48) | **<0.0001** |
| BAL eosinophils (%)* | 1.0 (0.15-3.90) | 0.6 (0.20-2.44) | 0.3556 |
| Tissue eosinophils (cells/mm^2^) | 133 (61.0-1654) | 268.5 (67.75-434.0) | 0.50 |

*BAL = Bronchoalveolar lavage, BMI = body mass index, COPD = chronic obstructive pulmonary disease, FEV 1 = forced expiratory volume in one second, FVC = forced vital capacity. Data are presented as median and IQR (interquartile range) unless otherwise indicated. Continuous data were analysed using a one-tailed Mann Whitney test; categorical data were analysed using a Fisher’s Exact test. #ICS dose data shown represents 9 COPD subjects with <300 cells/µl and 9 COPD subjects with ≥300 cells/µl. *BAL data shown represents 8 COPD subjects with <300 cells/µl and 10 COPD subjects with ≥300 cells/µl.*
